# Clinical and Molecular Aspects of C2orf71/PCARE in Retinal Diseases

**DOI:** 10.3390/ijms241310670

**Published:** 2023-06-26

**Authors:** Maddalen Zufiaurre-Seijo, José García-Arumí, Anna Duarri

**Affiliations:** Ophthalmology Research Group, Vall d’Hebron Institut de Recerca (VHIR), Vall d’Hebron Hospital Universitari, 08035 Barcelona, Spain; maddalen.zufiaurre@vhir.org (M.Z.-S.); jose.garciaarumi@vallhebron.cat (J.G.-A.)

**Keywords:** retina, retinitis pigmentosa, cone-rod dystrophy, *C2orf71* gene, PCARE, photoreceptors, cilia, ciliopathies, outer segment, RPE

## Abstract

Mutations in the photoreceptor-specific *C2orf71* gene (also known as photoreceptor cilium actin regulator protein PCARE) cause autosomal recessive retinitis pigmentosa type 54 and cone-rod dystrophy. No treatments are available for patients with *C2orf71* retinal ciliopathies exhibiting a severe clinical phenotype. Our understanding of the disease process and the role of PCARE in the healthy retina significantly limits our capacity to transfer recent technical developments into viable therapy choices. This study summarizes the current understanding of *C2orf71*-related retinal diseases, including their clinical manifestations and an unclear genotype-phenotype correlation. It discusses molecular and functional studies on the photoreceptor-specific ciliary PCARE, focusing on the photoreceptor cell and its ciliary axoneme. It is proposed that PCARE is an actin-associated protein that interacts with WASF3 to regulate the actin-driven expansion of the ciliary membrane during the development of a new outer segment disk in photoreceptor cells. This review also introduces various cellular and animal models used to model these diseases and provides an overview of potential treatments.

## 1. Introduction

The photoreceptor cilium actin regulator PCARE, formerly known as the chromosome 2 open reading frame *C2orf71* gene, was discovered for the first time when it was shown to be mutated in a subgroup of retinitis pigmentosa patients (RP54) (OMIM #613428) by homozygosity mapping [1,2]. PR54 is characterized by typical signs of RP, including poor night vision, peripheral visual field loss, retinal bone spicule-type pigment deposits, pale optic discs, and significantly reduced or absent responses on electroretinography. Atypical features observed include early degeneration of the cone photoreceptor system with macular abnormalities and ring scotomas on the visual field [2]. Moreover, patients may display an early-onset variant of cone-rod dystrophy (CDR or CORD23), with central vision loss and a ring scotoma around the fovea that proceeds to significant chorioretinal atrophy in the macular area [3]. Unfortunately, there is no cure for *C2orf71*-related RP54 and CRD. It is suggested that PCARE plays a role in actin remodeling and protein transport in the cilia of photoreceptors. The orthologues of *C2orf71*, *BC027072* (Gene ID: 225004) of Mus musculus [4], and *pcare1* of zebrafish (Gene ID: 100537793) [1] have been used as an alternative model to study the role of PCARE protein. Among the other findings, zebrafish *pcare1* expression starts to be detected simultaneously with the initiation of photoreceptor differentiation [1]. At the same time, mouse *BC027072* is expressed around embryonic day 14, demonstrating that the this gene plays a key role in the mature retina and might be relevant during retinal development [2].

In this review, we offer an overview of the current knowledge of retinal diseases caused by mutations in the *C2orf71* gene, RP54, and CRD, including clinical symptoms and mutation landscape, molecular and functional research on photoreceptor-specific cilium, cellular and animal models, as well as a summary of prospective therapeutic options. In addition to reviewing the present state of knowledge, we have sought to identify gaps in our understanding of the *C2orf71*/PCARE pathogenic mechanism and offered some directions for further research.

## 2. Relevant Sections

### 2.1. Retina and Photoreceptors

The retina, the innermost layer at the back of the eye, is the main structure responsible for converting light energy from photons into electric impulses (electromagnetic energy to electrochemical energy) sent to the brain via the optic nerve to form three-dimensional images [5]. The sensory tissue is divided into the neuroretina (NR) and the retinal pigmented epithelium (RPE). The neuroretina is a sensory layer formed by five major retinal neurons (photoreceptors, bipolar cells, amacrine cells, horizontal cells, and ganglion cells), Müller glial cells, astrocytes, microglia, and the retinal vascular cells. Among the retinal neurons, photoreceptor (PR) cells are specialized primary cilia [6,7]. The primary cilium differs from motile cilia since it acquires sensory roles. In this case, PRs capture and convert light into electrochemical signals in phototransduction [6]. RPE is an epithelial monolayer located between the NR and capillary bed. It has crucial roles in photoreceptor maintenance and function, such as phagocytosis of shed photoreceptor outer segments (OS) and nourishing and diffusing nutrients from the blood to photoreceptors, among others [8].

**Figure 1:**
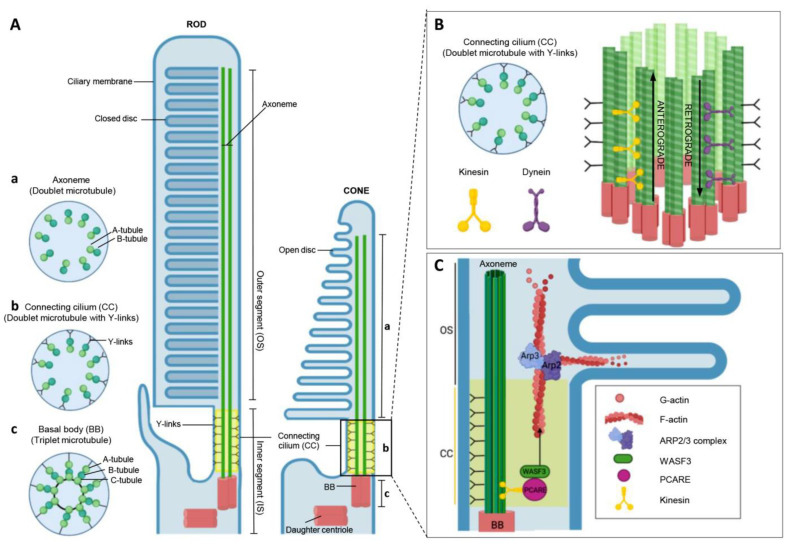
Photoreceptor structure, connecting the cilium and retinal function of PCARE. (**A**) The cross-sectional view of the microtubule structure changes from a triplet microtubule structure (basal body, BB) to a doublet with Y-links (transition zone, TZ, or connecting cilium, CC), ending in a doublet configuration in the axoneme. The primary cilium of photoreceptor cells (rod and cone) is formed by the axoneme and ciliary membrane, growing from the BB and elongating through the outer segment of photoreceptor cells. The TZ, or CC, is located between the BB and axoneme. The yellow box indicates the CC region. (**B**) Schematic representation of CC, where its microtubule configuration with Y-links, as well as the intraflagellar transport (IFT) molecular motors kinesin (anterograde) and dynein (retrograde), are shown. (**C**) The proposed role of PCARE in specialized photoreceptor cilium. PCARE is located in the connecting cilium (CC) and recruits WASF3 protein from the cytoplasm to the CC. The PCARE-WASF3 complex activates ARP2/3 and promotes the formation of F-actin. This will cause remodeling and expansion of the ciliary membrane expansion and give rise to a new OS disk. The figure was created with BioRender.com.

PRs are classified into two groups: rods and cones. They are morphologically and functionally distinct and express several light-sensitive visual pigments, opsins, and other proteins related to phototransduction or disk structure [6]. Rod photoreceptors contain rhodopsin, which functions in dim light and can detect one single photon. In contrast, cone photoreceptors contain cone opsins (short-, medium-, and long-wavelengths), which function in bright light and are responsible for color vision and acuity. PRs have four main structural and functional regions: the cell body, synaptic terminal, inner segment (IS), and OS (a highly modified primary cilium [1]) (Figure 1B). IS and OS are continuous through an extremely narrow connecting nonmotile cilium, a cylindrical stalk containing a modified cilium called the connecting cilium (CC) [9] (Figure 1C). In fact, the OS of the photoreceptor functions as a modified primary cilium. The cilium of OS originates from the CC, a structure that resembles the transition zone (TZ) in other cilia [10]. At the base of the CC lies the basal body (BB), whereas, at the distal end of the CC, it gives rise to the ciliary axoneme, which runs through all the OS. This CC has a bundle of 9 microtubule doublets (9+0 array of microtubules) becoming singlets near the tips [11], serving as (1) a conduit for large amounts of proteins, membranes, and small molecules, biosynthesized in IS, that must be displaced to the OS, and (2) as a diffusion barrier to prevent the flow of OS components to other parts [9,12].

### 2.2. Biogenesis of Primary Cilium

The cilium is formed by a microtubule-based backbone (the axoneme) surrounded by a ciliary membrane. The cilium can be distinguished as motile and nonmotile (primary cilia). Primary or sensory cilia are associated with sensing extracellular signals and controlling development cues, cell proliferation, and cell polarity [13]. Thus, photoreceptor cilia located in OS are classified as primary cilia since they can sense and transduce external signals in response to light energy [14] (Figure 1). Biogenesis of nonmotile cilia occurs from a mother centriole, which forms a basal body [15]. The mother centriole (MC) and daughter centrioles (DC) interact with pericentriolar material (PCM), such as the PCM1 protein, to form the centrosome (which nucleates, anchors, and organizes the microtubules). Afterward, MC, due to its apical migration, becomes the BB, which displays a triplet microtubule configuration (Figure 1A), where proteins such as coiled-coil and C2 domain-containing 2A (CC2D2A), CEP135, and SAS6 participate in its formation. Preciliary vesicles (PCVs) start to be developed in the Golgi and are transported to the distal part of the BB through myosin MYO5A, dynein, and the ARP2/3-associated branched actin network. These vesicles form a larger ciliary vesicle, transporting proteins relevant for the maturation of the BB and the nascent cilia. Docking of vesicles is followed by the recruitment of intraflagellar transport (IFT) complexes and motor proteins to the distal part of BB, proteins that will induce the formation of the ciliary axoneme. Since no known protein synthesis machinery exists in the cilia, the intraflagellar bidirectional transport system is crucial to delivering components to the cilia [10] (Figure 1B). First, the proximal part of the axoneme will be formed, known as the TZ. In the TZ, the microtubule configuration changes to doublets (9+0 configuration) (Figure 1A). It is cross-linked to the ciliary membrane by the Y-links, filamentous structures named Y-shaped links that connect the doublet microtubules to the ciliary membrane. TZ acts as a molecular barrier from the IS to the OS of photoreceptor cells, the CC, and serves as a passageway regulating the continuous flow of new proteins. Interactome analysis in primary cilia has pinpointed many protein complexes unique to the TZ that have since been reported in the CC that connects photoreceptors’ OS (Figure 1) [16,17]. Most of them are part of the Meckel (MKS) module (CC2D2A, B9D1, B9D2, MKS1, AHI1, TCTNs, and TMEM) and of the nephrocystin (NPHP) module (RPGRIP1, RPGRIP1L, NPHP1, and NPHP4, among others) associated with the Y-linker-residing protein CEP290 [18]. The inversin compartment (INVS, NEK8, NPHP3, ANKS3, and ANKS6) localizes outside the TZ. It communicates with the MKS and NPHP (Nephronophthisis) complexes through INVS, creating a connection between them [19,20,21]. After the TZ is developed, it gives rise to the axoneme (“9+0” configuration). The axoneme elongates into the OS, and the ciliary membrane extends in tandem with the transport of the ciliary proteins by ciliary trafficking machinery [16,22].

### 2.3. OS Disk Neogenesis

Due to the high oxidative stress suffered by OS, the OS disks must be continuously renewed [9]. Hence, 10% of rod photoreceptor old disks are shed daily at the apical OS tip, phagocytized, and absorbed by the RPE [20]. While shedding occurs, the IS synthesizes opsins and other membranous materials. In contrast, OS disks are generated by the distal end of CC, specifically where CC meets the base of the OS [21]. The disk formation occurs through the evagination and expansion of the ciliary plasma membrane by branched actin networks at the evagination site. This led to the idea that actin is critical to OS renewal [22]. Proteins such as ROM-1 and PRHP2 ensure the membranous disks’ unique design is maintained [23], whereas RP1, which binds axonemal microtubules, controls the membranous disks to stack correctly. Then, the proximal part of the CC will allow the passage of new disks into the OS. [24]. Afterward, disks are transported through the axoneme in the OS, a process catalyzed by myosins or kinesins (anterograde transport) [15].

Therefore, given the highly complex cilium of photoreceptor cells, mutations in ciliary genes may cause protein malfunction and incorrect localization and potentially impair the structure, biosynthesis, function, and maintenance of photoreceptor cilia, resulting in retinal ciliopathies. Multiple proteins, such as WASF3, PCARE (*C2orf71*), ARP2/3, and SPATA7, are linked with the TZ, the CC, and OS disk morphogenesis (Figure 1C). Mutations in genes expressed in the retina’s photoreceptor cells can cause inherited ocular disorders implicating a progressive loss of photoreceptor cells and loss of vision, such as RP or CRD [1,17,20,25,26,27,28,29].

### 2.4. Molecular Aspects of PCARE

*C2orf71* is a two-exon gene spanning a 12.5-kb region on 2p23.2 [30], where one of the exons contains 95% of the coding region [4]. This gene encodes a ciliary intracellular protein of 1289 amino acids and 140 kDa known as PCARE, a name proposed by Corral-Serrano et al. [12]. It is nearly exclusively expressed in the retina [2,4], specifically at the initiation site of OS disks. Unfortunately, the PCARE protein encoded by the *C2orf71* gene shows no homology with other known human proteins. Therefore, there is a lack of information on the primary sequence and its function [1,4,12,31,32]. Yet, comparing the PCARE protein sequence between mammals, birds, and fish vertebrate classes presents some well-conserved areas undergoing post-translational lipid modifications [1].

**Figure 2:**
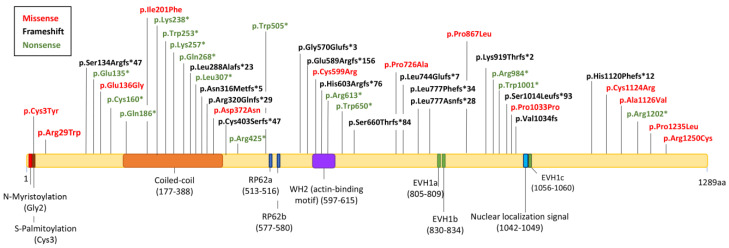
RP54 and CRD-associated mutations in PCARE protein. Schematic representation of missense (red), frameshift (black), and nonsense (green) mutations. In the PCARE structure, colored columns represent the functional motifs: N-myristoylation (Gly2), S-palmitoylation (Cys3), coiled-coil domain (aa 318–333), two RP62 kinase-binding motifs, a WH2 acting binding motif (aa 597–615), three proline-rich regions (EVH1 domain-binding motifs) (aa 805–809, 830–834, 1056–1060), and a nuclear localization signal (aa 1042–1049).

Identifying the functional domains in the PCARE protein could indicate its functions in the retina, and several authors have tried to predict its molecular aspects. Even if PCARE does not contain any highly conserved functional domains, Corral-Serrano et al. [12] achieved the detection of three relevant structural protein sequence motifs: PCARE fragment 1 (F1, N-terminal containing amino acids (aa) 2 to 449), fragment 2 (F2, aa 450 to 900), and fragment 3 (F3, C-terminal containing aa 901 to 1289) (Figure 2). The PCARE-F1 region shows post-translational lipid modifications. By the alignment of the first twenty-five amino acids of PCARE orthologs from different species, a complete conservation of the first three amino acids (methionine, glycine, and cysteine), along with the proline at position five and the serine at position six, was observed [1]. The glycine at position 2 (G2) suggests that this position could be myristoylated since it is a common modification in the G2 position of many proteins. Furthermore, S-palmitoylation is also found at cysteine at position 3 (C3). These post-translational lipid modifications are required for stable membrane anchoring, protein trafficking, and signaling [1,12]. PCARE-F1 also contains a likely structured domain, referring to the helical domain forming an antiparallel coiled coil from amino acids 171 to 388, relevant for ciliary trafficking. PCARE-F2 contains an actin-binding WH2 short-linear motif (SLiM), amino acids 597 to 615 [32], capable of binding to actin filaments. Moreover, this region contains two target sites (RP62a and RP62b) for the MAK/RP62 kinase, a photoreceptor cilium-associated protein crucial for ciliary length and retinal health [33]. It also has two enabled/VASP homology 1 domain (EVH1)-binding motifs in amino acids 805 to809 and 830 to 834 positions. Lastly, PCARE-F3 presents a predicted nuclear localization signal, with a proline-rich region from amino acids 1013 to 1095 and the third EVH1-binding motif from 1056 to1060 positions [1,12,32]. Proteins with EVH1 domains are associated with promoting actin filament formation since they bind to the proline-rich regions of other proteins implicated in actin dynamics. Proline-rich domains alone can have actin polymerase activity in the presence of profilin-actin, and the tandem of proline-rich domains and WH2 domains cooperates to enhance actin polymerase activity [32,34]. Additionally, Afanasyeva T et al. [32] and Corral-Serrano et al. [12], among others, studied the localization of these three fragments and the full-length protein PCARE in the photoreceptors. PCARE-F1 and the full-length protein are localized into the BB and axoneme of the cilium. At the same time, PCARE-F2 is present in the cytosol, and PCARE-F3 is mainly localized to the nucleus, even though it is also observed in the cilium axoneme [1,12].

### 2.5. The Function of PCARE in the Retina

The localization, structure, and protein–protein interaction relationship of PCARE have been key factors in determining its functions. PCARE has been localized predominately in three areas: the ciliary BB, the accompanying DC, and the distal part of the CC stalk, where the first OS membrane disk neogenesis occurs [12,32] (Figure 2). Protein–protein interaction studies also found that besides finding proteins associated with the centrioles of the centrosome and/or the ciliary BB and microtubule-associated proteins, there was a relevant interaction between PCARE and proteins linked to actin dynamics. This matches the predicted structure of an actin-binding motif in PCARE. Hence, this suggests that the PCARE protein could be implicated in photoreceptor actin-based processes.

One of the key proteins implicated in actin dynamics, which potentially interacts with PCARE, is the WAVE regulatory complex (WRC) member WAVE3/WASF3. While all WASF proteins are localized generally in the leading edge of lamellipodia, their expression differs; WASF1 and WASF3 are brain-specific, whereas WASF2 is ubiquitously expressed [35]. Specifically, the interaction between WASF3 and PCARE in the axoneme induces ARP2/3 complex activation. The ARP2/3 complex is responsible for the actin nucleating activity and the formation of the F-actin network required to initiate OS disk formation [36] (Figure 2). Studies with ectopically expressed PCARE in cultured ciliated cells demonstrated that the PCARE–WASF3 interaction induces (1) recruitment of WASF3 from the cytoplasm to the photoreceptor cilium; (2) PCARE activates a Rho GTPase RAC1, a WAVE complex activator, which leads to an activation of WASF3; (3) WASF3 possibly activates ARP2/3, enhancing the formation of the F-actin network; and (4) PCARE-WASF3 recruits F-actin and forms a ciliary membrane expansion. Recently, the interaction region between PCARE and WASF3 was suggested to be in the N-terminal of PCARE. During this process, WASF3, ACTN1 (α-actinin), and PCARE have been localized at the site of initiation of OS disk morphogenesis in the apical and base regions of the CC region. More specifically, PCARE has been detected around the microtubules of the apical region of CC and extends to the initial part of the OS base in mouse photoreceptors [12]. Furthermore, endogenous human PCARE is observed in the ciliary membrane expansion and WASF3 [12,32], and the mouse PCARE homolog is implicated in intraflagellar transport across the CC [4].

By using inhibitors of actin polymerization, Corral and colleagues’ study reaffirmed a significant expansion size reduction, impeding new disk generation [12,31]. This corroborates that actin polymerization caused by PCARE and WASF3 interaction is crucial to induced ciliary expansion. Additionally, Nager et al. demonstrated that actin has a key role in releasing ectosomes from the ciliary tip since inhibiting actin with cytochalasin D leads to an accumulation of uncleaved buds at the tips of cilia [37].

Aside from the WASF3 protein, PCARE also interacts with proteins associated with different cellular components. These include proteins related to the centrioles of the BB (OFD1, CEP290, and CEP250), microtubule-associated (DCTN1/p150-glued and DCTN2/p50 dynamitin, PCM1, NINL, and KNSTRN9), microtubule-based kinesin motors (KIF20A, KLC2, and KLC4), and other proteins implicated in actin dynamics, mostly members of the ARP2/3 complex that induces actin polymerization. Among these proteins, PCARE interacts specifically with the microtubule-associated proteins DCTN1, KIF20A, IFFO1, and PCM1 [12]. This suggests that PCARE may also be required to organize the cytoplasmic microtubule network and ciliary trafficking. Yet, there are still some potentially undiscovered amino acid motifs in PCARE that play a role in ciliary trafficking [32].

Hence, PCARE is localized in the retina, specifically in the CC of photoreceptors, and interacts predominately with WASF3, an actin modifier that activates the ARP2/3 complex. Its function is the polymerization of G-actin, creating a branched F-actin network. This last network leads to a membrane extension, which induces an expansion of the ciliary tip, promoting disk morphogenesis. Considering this evidence, the PCARE protein may regulate the initial development of OS disks by transporting actin-associated components to the base of photoreceptor OS [12].

### 2.6. Retinal Diseases Associated with Mutations in C2orf71/PCARE

Inherited retinal diseases (IRDs) represent a heterogeneous group of rare disorders caused by over 281 genes (Retnet, May 2023). The prevalence of monogenic IRDs is approximately 1 in 2000 individuals, affecting more than two million people worldwide [38]. These diseases can be classified based on whether they predominantly affect the rods (e.g., RP) and the cones (e.g., CRD) or cause a more generalized photoreceptor disease (e.g., Leber congenital amaurosis (LCA)). The variability of an IRD’s genotype and phenotype makes diagnosis difficult; additionally, many IRDs share clinical characteristics. The clinical appearance of RP, the most prevalent type of IRD, may resemble that of LCA or CRD. First, it exhibits rod cell failure followed by cone cell degeneration, and the retinal pigment epithelium (RPE) can also be primarily involved. However, IRD exhibits comparable symptoms at later stages, including significant retinal atrophy, severe retinal cell death, and permanent sight loss. On the other hand, many traits can be encoded by a single gene, as exemplified by the most common IRD-associated gene, ABCA4. This causes Stargardt disease, CRD, pattern dystrophy, or achromatopsia, in line with the strong association of biallelic ABCA4 mutations with cone-dominated symptoms that primarily impact the central retina [39]. Because of this variability in genotypes and phenotypes, most IRDs are untreatable, with only a few clinical management options currently available.

Because *C2orf71* is a ciliary gene, *C2orf71* gene-related retinal dystrophies such as autosomal recessive RP or CRD are considered ciliopathies. Ciliopathies can be caused by defects in the structure or operation of the primary ciliary components, such as the TZ or BB, or they can be caused by deficiencies in the movement of ciliary axoneme components or the transcriptional control of ciliogenesis [40], affecting several organs/tissues [41].

#### 2.6.1. Retinitis Pigmentosa Type 54

RP is a group of heterogeneous diseases and the most common form of hereditary retinal degeneration, with a worldwide prevalence of 1 in 4000 [42] and more than 60 causing genes. Initially, rod photoreceptor cells started to be dysfunctional, and subsequently, cone cells began to be affected. The pathology causes a progressive loss of rod photoreceptor cells and night vision. It is characterized by night blindness, macular atrophy, narrowed retinal arterioles, constricted visual fields, or tunnel vision. In most cases, the genetic trait of RP is presented as autosomal recessive (arRP) (50–60%), autosomal dominant (30–40%), X-linked (5–15%), sporadic, mitochondrial, or digenic inheritance patterns [43]. Even if the age of onset is highly variable, most RP forms start with night blindness in young adulthood, followed by mid-peripheral visual field loss and tunnel vision. Later, the central vision started to reduce, leading to complete blindness in some cases. Focusing on arRP, which accounts for 50–60% of all RP cases, is considered more severe since it has a younger age of onset and faster disease progression.

Our gene of interest is causative for RP54, one of the progressive subtypes of inherited autosomal recessive retinal dystrophies [44,45]. In 2010, Nishimura et al. [1] and Collin et al. [2] reported for the first time five families with mutations in *C2orf71* showing signs of typical RP54 (Table 1). The genetic alteration of the *C2orf71* in photoreceptors and the RPE [46] (although little is known about their contribution to pathomechanism) promotes, in the first stages, progressive atrophy of the rods. This loss of the rods presents itself as night blindness (nyctalopia) and a reduced visual field (tunnel vision). In the following phase, the cones begin to degenerate due to macular edema, leading to central vision loss. However, *C2orf71* mutations seem to be linked to severe RP and early cone involvement [2], emphasizing this gene’s critical role in healthy photoreceptor function. The loss of photoreceptor cells promotes multiple complications, such as the almost complete replacement of the degenerated neuroepithelium by neuroglia, thickening of the retinal vessels, and in advanced stages, fibrosis of the vessels, arterioles, and/or veins of the retina [47].

#### 2.6.2. Cone-Rod Dystrophy

Mutations in 14 ciliary genes, the most important of which are *C8orf37*, *RPGR*, and *RPGRIP1*, have been found in CRD patients so far, and several of these are involved in ciliary transport [25,26,48]. Mutations in *C2orf71* have also been observed to cause CRD, although with less frequency [3,49]. This pathology has a prevalence of 1–40,000 individuals [50]. Cone dysfunction occurs first, followed shortly after by a deterioration of rod function, causing early signs of acuity and color vision loss, followed by diminished peripheral vision. While RP and CRD have different rod and cone photoreceptor dysfunction orders, symptoms occasionally overlap. Consequently, disease diagnosis becomes more challenging. Yet, the clinical course of CRDs is usually more severe and rapid than that of RP, which leads to earlier blindness, and the age of onset is usually in early adult life [3,51]. Boulanger-Scemama et al. described two individuals with homozygous p.Trp650* and p.Arg984* mutations in the *C2orf71* gene (previously related to RP54). These individuals experienced CRD that presented predominant central involvement with severe bilateral macular atrophy, primary photophobia, and bilateral central vision loss [52]. They estimated that the prevalence in the cohort was 2.5% (2 out of 78 autosomal recessive or sporadic cases) (Table 1). This is consistent with research by Collin et al., who previously described two RP54 individuals with abnormal multifocal ERG recordings that suggested more impaired cone than rod function despite substantial loss to both photoreceptor functions [2]. Moreover, Tiwari et al. and Serra et al. reported two other cases with an early-onset form of CRD that presented compound heterozygous mutations in the *C2orf71* gene: p.Gly570Glufs*3 and p.Leu744Glufs*7 [53], and p.Leu288Alafs*23 and p.Cys599Arg [3], both in compound heterozygosity. Interestingly, a homozygous mutation for p.Gly570Glufs*3 was associated with RP54. Thus, the genetics and symptoms of *C2orf71* retinopathies are variable, making diagnosis challenging.

The current total number of *C2orf71* mutations reported in the Human Gene Mutation Database (HGMD) is 56 (33 missense and nonsense, 15 small deletions, 7 small insertions, and 1 small insertion/deletion; accession date: 5 May 2023) associated with non-syndromic retinal ciliopathies. However, we could only report 44 disease-causing variants in homozygosity or compound heterozygosity in 40 families or individual cases, of which 27 were missense and nonsense, 6 deletions, 7 insertions, and 4 duplications (Table 1; Figure 2). The difference in the number of mutations described in the HMGD and the reported pathogenic/likely pathogenic mutations lies in the additional variants, with only one hit in the *C2orf71* gene [30,54,55]. This is due to the highly polymorphic nature of the *C2orf71* gene and the sequencing methods used. We have collected additional data from 48 variants not included in Table 1 (except for p.Arg29Trp, p.Glu136Gly, and p.Asp372Asn), including 39 missense, 5 nonsense, 2 small duplications, and 2 small deletions (Supplementary Table S1). Some of these mutations were predicted to be potentially pathogenic or benign or found in 1000 Genome or Exome Variant Server databases with a frequency higher than the prevalence rate of RP (allelic frequencies of ≤0.5% were considered putatively pathogenic). Moreover, current sequencing strategies may not detect large heterozygous deletions, mutations in the 5′UTR, or intronic variants. These make genetic research on IRDs incredibly difficult, with many affected people (30–40%) remaining genetically unexplained.

**Table 1:** Genetic and clinical characteristics of patients with mutations in PCARE.

Family	Sex	Age	Type of Mutation	Allele 1	Protein	Allele 2	Protein	Clinical Phenotype	Refs.
1	-	-	(1) Missense (2) Frameshift	c.8G > A	p.Cys3Tyr	c.958_959insA	p.Arg320Glnfs*29	-	[56]
2	M	10	Missense	c.85C > T	p.Arg29Trp	c.3748C > T	p.Arg1250Cys	Ring-shaped macular hyper-fluorescence, EZ present in the fovea	[57]
3	F	63	(1) Frameshift (2) Nonsense	c.402_405del	p.Ser134Argfs*47	c.3604C > T	p.Arg1202 *	Large patches of hypo, retinal thinning	[39,58,59]
4	F	14	Missense	c.403G > T	p.Glu135 *	c.403G > T	p.Glu135 *	Small atrophic spots grouped in the foveal area	[60]
5			Missense	c.407A > G	p.GLu136Gly	c.3704C > T	p.Pro1235Leu	Peripherally speckled, Retinal thinning, puckering	[54]
6	F	20	Missense	c.478_479insA	p.Cys160 *	c.478_479insA	p.Cys160 *	Outer retinal dystrophy with thinning of the photoreceptor layers, mid-peripheral fundus atrophy, and some bony spicules	[59]
7	M	39	Nonsense	c.556C > T	p.Gln186 *	c.556C > T	p.Gln186 *	Poor night vision, peripheral bone-spicule-type pigment deposits, attenuation of retinal blood vessels, severe retinal atrophy, and pale appearance of the optic disk. Myopic since childhood	[2]
	M	37	Nonsense	c.556C > T	p.Gln186 *	c.556C > T	p.Gln186 *		
	M	25	Nonsense	c.556C > T	p.Gln186 *	c.556C > T	p.Gln186 *		
	F	-	Nonsense	c.556C > T	p.Gln186 *	c.556C > T	p.Gln186 *		
8	M	18	Missense	c.601A > T	p.Ile201Phe	c.601A > T	p.Ile201Phe	Pale optic disc, retina vessels attenuation, bone spicule pigmentation, macular unstructured, atrophy in left macula	[1,61]
9	M	31	Nonsense	c.712A > T	p.Lys238 *	c.712A > T	p.Lys238 *	RP	[62]
10	F	26	Nonsense	c.759G > A	p.Trp253 *	c.759G > A	p.Trp253 *	Perifoveal ring, central mottling atrophy ODS, photoreceptor and retinal layer structure loss	[1]
	F	17	Nonsense	c.759G > A	p.Trp253 *	c.759G > A	p.Trp253 *	Perifoveal ring, central mottling atrophy ODS, Vitelliform lesion subfoveal, photoreceptor layer loss	
	M	26	Nonsense	c.759G > A	p.Trp253 *	c.759G > A	p.Trp253 *	Foveal sparing, hyper ring macula, hypo background, and many nummular atrophic areas ODS, photoreceptor loss with abnormal retina lamination	
	M	41	Nonsense	c.759G > A	p.Trp253 *	c.759G > A	p.Trp253 *	Extensive widespread hypo, few small hyper areas throughout ODS	
	M	20	Nonsense	c.759G > A	p.Trp253 *	c.759G > A	p.Trp253 *	Extensive widespread hypo, few small hyper areas throughout ODS, photoreceptor loss with a chaotic retinal structure	
	F	42	Nonsense	c.759G > A	p.Trp253 *	c.759G > A	p.Trp253 *	Macular and mid-peripheral speckled, retinal thinning, puckering	
	M	-	Nonsense	c.759G > A	p.Trp253 *	c.759G > A	p.Trp253 *	Earlier-onset and severe generalized dystrophy (<5 years) associated with nystagmus.	
	M	-	Nonsense	c.759G > A	p.Trp253 *	c.759G > A	p.Trp253 *	-	
11	M	48	Nonsense	c.769A > T	p.Lys257 *	c.769A > T	p.Lys257 *		[63]
12	M	15	(1) Nonsense (2) Frameshift	c.802C > T	p.Gln268 *	c.2756_2768del	p.Lys919Thrfs*2	Perifoveal speckled, retinal thinning	[59]
13	F	38	(1) Frameshift (2) Missense	c.860dup	p.Leu288Alafs*23	c.1795T > C	p.Cys599Arg	CRD—severe retinal dysfunction with marked macular atrophy	[3]
14	F	42	Nonsense	c.920T > A	p.Leu307 *	c.920T > A	p.Leu307 *	Severe peripheral atrophy with pigment, macular, and mid-peripheral speckled hypo/ hyper-AF	[59]
16	F	32	Frameshift	c.946 del	p.Asn316Metfs*5	c.946 del	p.Asn316Metfs*5	RP—night blindness, peripheral bone spicules, and attenuated retinal vessels, but evidence of early degeneration of the cone photoreceptor system	[2]
	F	31	Frameshift	c.946 del	p.Asn316Metfs*5	c.946 del	p.Asn316Metfs*5		
	M	37	Frameshift	c.946 del	p.Asn316Metfs*5	c.946 del	p.Asn316Metfs*5		
	M	30	Frameshift	c.946 del	p.Asn316Metfs*5	c.946 del	p.Asn316Metfs*5		
17	M	18	(1) Frameshift (2) Nonsense	c.946del	p.Asn316Metfs*7	c.3002G > A	p.Trp1001 *	Severe retinal dysfunction with marked macular atrophy.	[39,44]
18	M	49	Frameshift	c.947del	p.Asn316Metfs*7	c.1709_1728del	p.Gly570Glufs*3	RP	[53]
19	F	22	Frameshift	c.1206_1207dup	p.Cys403Serfs*47	c.1206_1207dup	p.Cys403Serfs*47	Macular and mid-peripheral speckled, retinal thinning, puckering	[59]
20	M	47	Nonsense	c.1273C > T	p.Arg425 *	c.3002G > A	p.Trp1001 *	Macular and nasal patchy hypo, retinal thinning, puckering	
21	M	45	Nonsense	c.1273C > T	p.Arg425 *	c.1514G > A	p.Trp505 *	Childhood. Bilateral retinal vascular attenuation, bone spicule-like pigmentation in the mid-periphery retina, RPE degeneration, pale optic discs.	[64]
22	M	25	Frameshift	c.1709_1728del	p.Gly570Glufs*3	c.1709_1728del	p.Gly570Glufs*3	Macular large hypo area, speckled to mid-peripheral, retinal thinning	[53]
	F	50	Frameshift	c.1709_1728del	p.Gly570Glufs*3	c.1709_1728del	p.Gly570Glufs*3	Retinal thinning, ORT	
23	M	13	Frameshift	c.1709_1728del	p.Gly570Glufs*3	c.2227_2228del	p.Leu744Glufs*7	CRD—Moderately heterogeneous macula, periphery normal, retinal thinning, loss of IS/OS	
	M	22	Frameshift	c.1709_1728del	p.Gly570Glufs*3	c.2227_2228del	p.Leu744Glufs*7	CRD—Heterogeneous macula, periphery normal, retinal thinning, loss of IS/OS	
24	F	-	Frameshift	c.1764del	p.Glu589Argfs*156	c.1764del	p.Glu589Argfs*156	RP	[65]
	F	-	Frameshift	c.1764del	p.Glu589Argfs*156	c.1764del	p.Glu589Argfs*156	RP	[44]
25	F	12	Missense	c.1795T > C	p.Cys599Arg	c.1795T > C	p.Cys599Arg	Night blindness, visual field constriction, decreased visual acuity, photophobia, abnormal color, and vision Pale optic nerve disks narrowed blood vessels, and bone spicule pigmentation in the periphery	[66]
26	F	24	Nonsense	c.1837C > T	p.Arg613 *	c.1837C > T	p.Arg613 *	-	[67]
27	F	34	(1) Nonsense (2) Frameshift	c.1837C > T	p.Arg613 *	c.3358_3359del	p.His1120Phefs*12	Macular speckled, retinal thinning	[59]
28		-	Nonsense	c.1949G > A	p.Trp650 *	c.1949G > A	p.Trp650 *	CRD—primary photophobia and central vision loss associated with predominant central involvement in autofluorescence imaging	[52]
29	F	8	Nonsense	c.1949G > A	p.Trp650 *	c.1949G > A	p.Trp650 *	Retinal thinning	
30	M	28	Frameshift	c.1979_ 1982delGCAA	p.Ser660Thrfs*84	c.1804_ 1805delAG	p.His603Argfs*76	-	[28]
31			Missense	c.2176C > G	p.Pro726Ala	c.3377C > T	p.Ala1126Val	-	[54]
32	M	20	Frameshift	c.2327dup	p.Leu777Phefs*34	c.2328_2344del	p.Leu777Asnfs*28	Perifoveal and mid-peripheral speckled, retinal thinning	[59]
	M	14	Frameshift	c.2327dup	p.Leu777Phefs*34	c.2328_2344del	p.Leu777Asnfs*28	Foveal sparing hypo, speckled periphery, spared mid-periphery, retinal thinning	
33	M	Early childhood	(1) Frameshift (2) Nonsense	c.2327dup	p.Leu777Phefs*34	c.2950C > T	p.Arg984 *	Foveal sparing hypo, speckled periphery, spared periphery, retinal thinning, ORT.	[44]
34	F	6	Frameshift	c.2756del13	p.Lys919Thrfs *	c.2756del13	p.Lys919Thrfs *	Peripheral-field loss, extinct ERGs, and bone-spiculated pigmentation in the peripheral retina	[2,39]
	M	47	Frameshift	c.2756del13	p.Lys919Thrfs *	c.2756del13	p.Lys919Thrfs *		
	M	59	Frameshift	c.2756del13	p.Lys919Thrfs *	c.2756del13	p.Lys919Thrfs *		
35	M	-	Frameshift	c.2756del13	p.Lys919Thrfs *	c.2756del13	p.Lys919Thrfs *		
36			Nonsense	c.2950C > T	p.Arg984 *	c.2950C > T	p.Arg984 *	RP—Extensive chorioretinal atrophy, retinal thinning of the foveal region and choroidal hyperreflectivity by window defect, “speckled” AF, granular pattern, optic disc pallor extending beyond the vascular arcades without peripapillary sparing. CRD- primary photophobia and central vision loss associated with predominant central involvement	[44,52]
37	M	32	Nonsense	c.3002G > A	p.Trp1001 *	c.3002G > A	p.Trp1001 *	Macular and mid-peripheral speckled, retinal thinning, forming ORT	[59]
38	M	25	(1) Missense (2) Frameshift	c.3039dupC	p.Ser1014Leufs*93	c.1804_ 1805delAG	p.His603Argfs	Early-onset of CRD, well-circumscribed ring-shaped area of choroidal and RPE atrophy surrounding the fovea in the left eye and small white patches of atrophy around the fovea in the right eye	[3,39]
39			(1) Frameshift (2) Missense	c.3099_ 3100insCAGG	p.Val1034fs	c.3099T > C	p.Pro1033Pro	RP	[68]
40	F	49	Missense	c.3370T > C	p.Cys1124Arg	c.2600C > T	p.Pro867Leu	-	[28]

* Clinical phenotypes without specific disease specifications are RP. AF, autofluorescence; EZ, ellipsoid zone; ODS, right and left eye; ORT, outer retinal tubulation; RP, Retinitis pigmentosa; CRD, cone-rod dystrophy; -, not available.

### 2.7. C2orf71-Related Disease Clinical Features

A study showed that because cilia play a crucial role in photoreceptor function, patients with RP ciliopathy exhibit more severe loss of ellipsoid zone line width and short-wavelength autofluorescence ring constriction than RP patients without ciliopathy [28]. The range of abnormalities visible on the fundus appearance in RP54 includes chorioretinal changes and early-onset maculopathy. Even in the latter stages of the disease, retinal layer atrophy with preserved laminar structure and microstructural loss of photoreceptor integrity point to a primary photoreceptor disease process. Several reported patients had the degenerative sign of outer retinal tubulation in the area of the less severely damaged central macula or at the ‘‘junctional zone’’ of advanced to less-advanced disease severity. However, *C2orf71* patients (or siblings) with the same disease-causing genotype suffer from a different RP phenotype, other IRD, or variability in disease severity with unclear correlation, in contrast to other autosomal dominant RP cases, in which there is a clear correlation between genotype and phenotype (e.g., GUCY2D [69]). The rapid and severe retinal degeneration caused by *C2orf71* mutations occurs at an early age of onset [31]. While visual decline typically starts in the second or third decade, some functional vision can still be maintained into the fifth or sixth decade [59]. Thus, the age of disease onset and the position of the *C2orf71* mutation have no significant correlation. Moreover, although studies found no ethnicity relationship with RP54, different groups have different frequencies of *C2orf71* mutations and associated symptoms. RP54 is more prevalent in the Swiss [53] and Chinese [64] populations since the frequency of pathogenic sequence variations in *C2orf71* is 15% and 8%, respectively. In contrast, in the French [44] and Italian [39] populations, only 1% of patients present signs of RP54.

Depending on the mutated region, PCARE protein structure, function, and localization will be altered differently. Corral et al. [70] indicated that most patients contain mutations in the N-terminal of PCARE. However, after collecting all the identified variants, we found that 61% are located in the N-terminal (Figure 2).

Afanasyeva et al. [32] studied how the inactivation of the PCARE-relevant motifs affects their functions and localization. PCARE with an inactivated helix domain has been observed to change its localization from the cilia to the cytosol. Hence, the transport of WASF3 into the cilia will be disrupted. Moreover, the inactivation of EVH1 domain-binding motifs and myristoylation on the third cysteine impede the modification of the actin filament network in the ciliary tip. Dysfunction of the MAK/RP62 union domain causes elongated cilia, rhodopsin accumulation, and photoreceptor degeneration. Lastly, alterations in glycine 2, where myristoylation occurs, or Cys3, where palmitoylation happens, do not affect ciliary trafficking. Yet, it seems that membrane anchoring, and hence the ability of PCARE-WASF3 to reach the ciliary tip, is reduced when palmitoylation is affected. Other studies, such as Audo et al. [44], observed that a frameshift mutation (c.946delA, p.Asn316Metfs*7) located around the PCARE-F1 region induced a truncation of more than 80% of the PCARE protein. This truncation led to the creation of a dysfunctional PCARE protein, which hampered the neogenesis of the OS disks. Overall, any mutation that affects the relevant motifs showed a decreased frequency of ciliary tip expansion, which hinders the disks’ neogenesis and leads to photoreceptor dysfunction. Yet, Corral studies [70] demonstrate no correlation between the position and age of onset or the severity of symptoms.

### 2.8. Other C2orf71-Related Pathologies

Besides RP54 and CRD, the *C2orf71* gene has been found to induce other pathologies in combination with other genes. Digenic RP has been described for heterozygous PRPH2 and ROM1 mutations [71]. Similarly, Schorderet et al. identified a heterozygous p. Arg571-Pro576del mutation in *C2orf71* and a heterozygous p.Pro231Ser mutation in FSCN2 to cause RP [72]. In another case, a CEP250 homozygous nonsense mutation (p.Arg1155*) in combination with a heterozygous null *C2orf71* allele (p.Gln1097*) was found to be associated with atypical Usher syndrome. This syndrome is characterized by early-onset sensorineural hearing loss and mild RP [73]. Additionally, Y. Liu et al. found a heterozygous nonsense mutation in *C2orf71* (p.Ser512*) in combination with a complex heterozygous protein-truncating mutation in RP1L1 (p.Lys111Glnfs*27 and p.Gln2373*), both on the maternal allele. This was observed in a single case with atypical RP, hearing loss, ataxia, and cerebellar atrophy [45]. As a consequence of this mutation, retinal disorganization with a severe cerebellar defect was observed in the zebrafish model.

By conducting an association analysis between *C2orf71* SNPs and colorectal cancer risk in 1419 Chinese Han participants, Jiang et al. found several SNPs (*C2orf71*-rs17744093, -rs10200693, -rs10166913, -rs17007544, and -rs13385188) were associated with increased colorectal cancer risk [74].

### 2.9. Animals Used for Studying C2orf71/PCARE Mutation

The absence of any primary structural information and the lack of an animal model present a significant impediment to understanding the function of this protein and its relationship to retinal disease in humans. So far, only two animal models of PCARE have been developed: two models of the zebrafish *C2orf71* as well as knockouts of the C57BL/6J mouse *C2orf71* homolog (*BC027072* or BC^−/−^) [1,4,31].

Zebrafish represent an ideal model system to study IRDs because (1) zebrafish embryos develop externally and are transparent, so the development of the eye can be monitored more easily; (2) their rapid development facilitates studies of visual responsiveness; (3) their amenability to genetic manipulation; and (4) they are diurnal animals, similar to humans, with a fully laminated and light-responsive retina 72 to 96 hours post-fertilization, besides having a cone-rich retina, which is beneficial to studying cone diseases. Zebrafish contain duplicate genes, including *C2orf71* (*pcare1* and *pcare2*), but mutant and knockdown models of the *pcare1* zebrafish have only been used [1,31]. The *pcare1* zebrafish resulted in an early-onset alteration: thinning of the photoreceptor outer segment layer and an altered behavioral response to light, a visual dysfunction. These suggest that PCARE is involved in photoreceptor OS maintenance [1,31]. Nonetheless, the zebrafish models present some notable differences from humans. Because it contains duplicate genes, even if disruption of *pcare1* causes retinal degeneration, *pcare2* is still functional and might also be implicated in outer segment disk formation. Thus, decreasing or abolishing the expression of *pcare1* does not entirely mimic the retinal phenotype of humans with mutated *C2orf71*. Zebrafish can also regenerate the retina, which limits the retinal degeneration caused by this mutation. In addition, the knockdown model is not useful for studying the long-term effects of mutations [1].

Using mouse models in IRDs is important since mice and humans share 79% of the amino acid sequence identity of proteins encoded by genes implicated in IRDs, including PCARE [75]. Its accelerated life span and generation time allow us to analyze the progression of eye disease in a short period of time. Moreover, due to their similarity to human physiology and anatomy, this model has also helped to design therapies to interfere with the progression of the disease [76]. Kevany et al. generated a knockout of the mouse *C2orf71* homolog, *BC027072 (BC^−/−^)*. Mouse PCARE consists of 1279 amino acids. The *BC^−/−^* mice show severe retinal dystrophy with early onset (8 weeks), similar to human patients. Knockout of BC displays a thinning of the ONL and a highly disorganized OS, and around the age of 6 months, it presents almost a complete degeneration of the ONL. On the other hand, this mouse model also contains some disadvantages; in contrast to the zebrafish model, mice are nocturnal animals, and therefore, cones are spread throughout the retina instead of having a cell-rich region such as a human fovea. This last shortcoming challenged the attempt to mimic cone disorders in a mouse model. Moreover, the CC of cones is surrounded by a periciliary membrane complex, while humans are shrouded by calyceal processes, which might affect the ciliary transport process [4,12]. The demonstration that loss of function of the zebrafish ortholog and mouse ortholog leads to early-onset alterations in visual behavior suggests that PCARE is likely implicated in the development of vision.

Since the beginning of the 20th century, dogs have been known to suffer from a complicated series of genetic retinal dystrophies called progressive retinal atrophy (PRA). These conditions lead to the degeneration of rod and cone photoreceptors, which impairs vision [77]. So far, six dog breeds, including the Gordon and Irish Setters [78], Standard Poodle and Tibetan Terrier [79], Polski Owczarek Nizinny [80,81], and Old Danish Pointing Dogs [82], have been linked to the c.3149_3150insC variation in *C17H2orf71* (Canis lupus familiaris, *C2orf71* human homolog) and the development of autosomal recessive rod-cone dysplasia 4, a type of PRA. Although these dogs are not animal models to study the disease pathophysiology, they could represent a feasible natural-occurring model to help develop new treatments.

### 2.10. In Vitro Models for C2orf71/PCARE Studies

Almost all cells produce a primary cilium, which either acts as a sensor of the environment or as a precursor to a group of motile cilia. Thus, primary ciliopathies are caused by structural or functional defects in the primary cilium [41]. Functional studies in animal models have partially revealed the pathophysiological mechanisms connecting ciliary gene mutations to the observed phenotypes. Still, the development of novel therapeutic approaches for primary ciliopathies has previously been constrained by the absence of reliable human cell models [83].

The ability to create an infinite number of tissue-specific progenitor cells or terminally differentiated cells is provided by induced pluripotent stem cell (iPSC) technology, which enables the reprogramming of somatic cells into a pluripotent state [84]. This approach offers a perfect in vitro model to investigate the pathophysiology of a specific IRD for disease modeling and treatment development by contrasting normal and patient-derived cells. Derived from human PSCs, retinal organoids are valuable as in vitro models for retina formation because they are three-dimensional structures that mimic the spatial and temporal differentiation of the retina [85,86]. Staining retinal organoids with PCARE and WASF3 at different developmental stages, Corral-Serrano et al. found that endogenous PCARE started to be expressed at day 120 of differentiation in the photoreceptor cilium. From day 150 on, the PCARE expression at the cilium’s tip increased and was more prominent when the tip began to expand to form the disc expansions around day 180 [12]. However, working with hPSC is expensive and time-consuming. An alternative approach is in vitro transdifferentiation, or direct reprogramming. Direct reprogramming is a promising, easy, and affordable method for producing target cells from somatic cells without using iPSC. Komuta et al. [87] transduced peripheral blood mononuclear cells with Sendai viral vectors that expressed CRX. This approach resulted in the generation of photoreceptor-like cells. They showed that retinal disease-related genes, essential for photoreceptor activity, were effectively expressed, including *C2orf71*.

On the other hand, RPE is crucial for photoreceptor development and function. Since the primary cilium is necessary for the complete maturation of the RPE, it is known that RPE maturation defects in ciliopathies occur before photoreceptor degeneration [88]. PSC-RPE obtained from knockdowns of ciliary-trafficking proteins and patients with ciliopathy exhibit immaturity, impaired development of apical processes, diminished functioning, and decreased expression of adult-specific genes [88,89]. Although it has been shown that ciliary gene mutations in RPE are linked to ciliary defects and retinal diseases, the exact causal relationship and underlying mechanisms by which primary cilia cause RPE-related diseases are still unknown. Corral-Serrano et al. and Nishimura et al. showed that when PCARE was overexpressed in hTERT RPE-1 cells, WASF-3 moved from the cytoplasm to the cilium. This movement occurred in the presence of identifiable actin filaments, leading to the expansion of the ciliary membrane, forming a bulbous tip [1,12]. The formation of the new outer segment discs by such expansions was demonstrated by retinal organoid culture. The production of the ciliary tip expansions was likewise inhibited by cytochalasin D or latrunculin B treatment and by siRNA-mediated ARP2 knockdown in mIMCD3 cells [12]. Similar to how wild-type PCARE had the exact same location as the RP-causing mutant but failed to expand the ciliary tip to the same extent, this was thought to be due to a lack of actin remodeling [12]. Hence, PCARE is a protein specific to the retina that may enable actin remodeling by enlisting WASF3 to cause membrane expansion, resulting in the formation of new outer segment discs for photo-sensing.

### 2.11. Therapies to Treat IRDs

Research on retinal ciliopathies and their therapies is important since genes linked to photoreceptor cilia shape and/or function account for almost 25% of all retinal degenerative diseases [15]. Tremendous efforts have been made to develop various treatments to prevent or treat retinal disorders. Specifically, in RP, CDR, and LCA caused by mutations in ciliary genes leading to incorrect protein expression, restoring normal gene expression via various approaches, such as gene therapy, might be a potentially helpful treatment strategy [26]. Other authors have extensively reviewed a general description and a discussion of advanced therapies to treat IRDs [90,91,92,93,94]. Here, we only provide a summary of the actual treatments.

The prognosis of RP is challenging due to the variability of gene mutations. The disease’s progress might differ based on mutations in a particular gene and other factors. According to research studies, symptoms can appear at any age, with an average of 4–12% yearly progression in losing the visual field. Unfortunately, there is presently no cure for RP54 or other IRDs. Most RP patients use traditional therapies, including retinoids, vitamin A supplements, sun protection, visual aids, and medical and surgical operations to address ocular comorbidities, which only work to halt the disease’s development but do not cure it. Given the restricted therapeutic landscape, creating novel and customized therapy modalities that target retinal degeneration is essential. Identifying innovative molecular treatments targeting particular receptors or pathways will lay the groundwork for modern drug development. Currently, 131 drugs are being developed for RP, with 30 in late-stage clinical development (24 in phase II and 6 in phase III studies) and 80 in preclinical development [94,95]. In clinical practice, the stage of the disease will probably play a role in administering the best therapeutic type. Overall, therapeutic strategies that aim to save photoreceptors are more likely to be used earlier in the disease. Gene therapy (including the CRISPR-Cas gene editing system, RNA replacement, antisense oligonucleotides, etc.), neuroprotection, and antibody therapies are more effective when used earlier in the disease course because they need the presence of (non-functional) endogenous photoreceptor target cells [96]. The goal is to use a gene complementation strategy in recessive RP, which manifests a loss of function of the target protein, and the most promising method to date to stop photoreceptor cell loss in retinal disorders is gene delivery using AAVs. Gene therapy methods for dominant RP include gene suppression with or without gene complementation. For recessive RP, the best example is the only available gene therapy—Luxturna (voretigene neparvovec)—developed for a specific gene, *RPE65*, which can only be given to a limited subset of individuals. So far, 31 patients have received Luxturna. After one year, there is a considerable improvement in visual function, and no significant adverse events occur. The improvement remains after three to four years of follow-up [97,98]. In addition, several RP-associated genes, including *RPGR* [99], *GUCY2D*, *XLRS*, and *CRB1*, are now targeted in gene therapy studies [91]. Although not for PCARE, other preclinical therapies targeting retinal ciliary genes are being investigated that could shed light on a potential treatment for *C2orf71* retinopathies. AAV-based *LCA5* and *NPHP5* gene augmentation resulted in photoreceptor cell survival, preservation of OS axoneme structure, and partial restoration of the bulge area, important for the OS disc formation and relocation of rod and cone opsins [100,101,102]. AAV-based *PRPF31* gene therapy can correct RPE’s ciliary and phagocytosis abnormalities, offering RP patients a viable treatment option [103]. However, the AAV virus cannot deliver large genes such as CEP290 because of its packing limit of 4.7 kb. Although various AAV strategies have been employed, such as dual or triple AAV vectors, antisense oligonucleotides, or CRISPR/Cas9-based strategies, their effectiveness has not yet been shown [104,105]. Another drawback is that many patients do not meet the criteria for gene therapy. Alternatively, identifying potential drug candidates—not based on specific genes—to improve photoreceptor survival is a promising and broader option. For instance, Chen et al. found that Reserpine, a Rauwolfia alkaloid, effectively improved cilium assembly and photoreceptor survival by partially restoring proteostasis [106,107].

Thus, these strategies might not be suitable for treating severe, end-stage retinal degeneration. Therefore, when photoreceptor degeneration is progressing, patients, regardless of genotype, may benefit from cell therapies (reviewed in [93]), optogenetics [108], retinal prosthesis [92], and photo-switchable chemicals [109].

Patients are more likely to receive a diagnosis at a late stage of the disease due to frequent referral delays. By this point in the disease, visual acuity has been significantly compromised, and visual field problems have worsened. End-stage RP is anticipated to be the first area in which regenerative medicines will focus on cell therapy. Photoreceptor or RPE cell transplantation as a replacement therapy has been shown to be a potential approach to regenerating diseased retinas [93]. Material transfer between engrafted photoreceptors and the host retina can also contribute to the positive effects of photoreceptor transplantation. This is particularly significant when photoreceptors are still not fully functioning because of a lack or reduced expression of crucial proteins [110,111,112]. It is nonetheless remarkable that graft photoreceptor cells can also have a neuroprotective impact, in part by secreting helpful substances.

The optogenetic approach involves the delivery and induction of light-responsive channels, such as opsin, in the host’s remaining retinal ganglion cells or bipolar cells, stimulating light and invoking a visual response [108]. Optogenetic treatment for RP patients is now being tested in many human clinical studies (NCT02556736, NCT03326336, NCT04919473, and NCT04278131).

Electronic retinal implants are intended to provide severely vision-impaired people with a rudimentary sensation of visual function, but the benefits are currently modest. Basically, retinal prostheses use an electrode array to provide electrical pulses to the surviving retinal nerve cells. Both direct electrical stimulation and photodiode arrays, which are implanted directly into the retinal space and translate projected light patterns into local electric currents, can be used to operate retinal prostheses. Direct electrical stimulation involves an external processing unit (such as a digital camera mounted on eyeglasses) taking real-time images and transmitting them to the retinal implant. Over the past 20 years, several retinal implants have been created, but only three have received regulatory approval and have been implanted in more than 500 patients [92].

The many vision-restoration methods currently being researched or used in clinical settings each have shortcomings and translational difficulties [109,113]. Chemical photoswitches (such as spiropyrans, diarylethenes, fulgides, azobenzenes, naphthopyrans, and stilbenes) can be used to photosensitize retinal neurons in a blind retina devoid of photoreceptors. This approach can help overcome some of these difficulties. These compounds are still in the preclinical stage. Although preliminary trials are encouraging, they must be carefully assessed, and their therapeutic relevance will be demonstrated once their safety is confirmed.

### 2.12. Perspectives and Future Directions

In this review, we discussed C2orf71/PCARE-related human retinal ciliopathies and the respective models, focusing on their function, localization, and interactors. We have emphasized the importance of PCARE localization into multiple cilia compartments, where several proteins express differently, frequently interacting with proteins from neighboring or similar compartments. A consistent examination of tissue expression patterns is essential for determining the significance of this gene in the retina and other relevant cell types and whether similar mechanisms and signaling pathways are involved. A comparative functional investigation of distinct pathogenic mechanisms will be possible by standardizing the data acquired for each mutation. At various stages of RP54 or CRD degeneration, the histology of the retina, with a focus on the photoreceptor layer, must be documented. Electron microscopy in longitudinal and cross-sectional sections of the junction between the inner and outer segments might be useful in identifying structural defects induced by PCARE mutations. Understanding how mutations in this particular ciliary gene lead to diverse clinical symptoms is required for linking clinical diagnosis with molecular identification. Moreover, establishing better *C2orf71* patient-derived in vitro models, such as human retinal organoids, would open new approaches to studying relevant pathogenic mechanisms and structural defects and offer the possibility of developing novel and efficient therapies. Still, some questions remain. For instance, what factors contribute to variation in genotype–phenotype correlations? Why is there this variability in the age of onset? May the PCARE protein have other tissue-specific functions? And if so, why is it not described as syndromic RP or CRD? Targeting the photoreceptor cilium and ciliary genes may be a possible strategy for the treatment or prevention of retinal ciliopathies, given the important function of the photoreceptor cilium and the significant number of ciliary gene mutations that have been found in various retinal disorders. Particularly for gene augmentation and antisense-oligo nucleotide-based therapies, considerable progress has been made in refining the drug formulation and delivery techniques. Today, more research is being conducted on drugs targeting the proteostasis network to create more broadly applicable cures.

## 3. Conclusions

Despite substantial progress in understanding the pathological mechanisms of *C2orf71*-associated retinopathy, some areas still need to be addressed in the future. The frequency of *C2orf71*-associated retinopathy is unknown despite its frequent occurrence in some ethnicities. Due to the significant clinical and genetic variability, the different ages of onset, and the (yet) insufficient genetic data, it is challenging to assess the exact prevalence of *C2orf71*-related retinopathies. More emphasis should be placed on the sequence analysis and sequencing techniques of *C2orf71*-associated retinopathy cases from other populations to appreciate the allelic heterogeneity of these diseases and better understand the differences in the genetic background that may influence the manifestation of this disease. The broad functional range of *C2orf71* variations, from benign to pathogenic, leaves numerous *C2orf71* variants with unknown penetrance. This has a significant impact on genetic testing and patient genetic counseling. Because research studies on C2orf71/PCARE are limited, more functional research based on animal models and in vitro data can produce valuable outcomes to assess the pathogenicity of each PCARE variant in certain circumstances. Still, more functional tests are essential to understanding the variants’ consequences and their correlation with genetic and clinical studies. Understanding the fundamental genetic and molecular mechanisms of *C2orf71*-related retinopathies will allow us to build ‘personalized’ therapeutics to delay or stop disease progression.

## Data Availability

No new data were created or analyzed in this study. Data sharing is not applicable to this article.

## Supplementary Materials

The following supporting information can be downloaded at: https://www.mdpi.com/article/10.3390/ijms241310670/s1. References [114,115,116] are cited in the Supplementary Materials.
