# Supplementary material for: Clinical and Molecular Aspects of C2orf71/PCARE in Retinal Diseases
Source: Int J Mol Sci. 2023 Jun 26;24(13):10670. doi: 10.3390/ijms241310670 (PMC10341768; doi:10.3390/ijms241310670)
Supplement: Supplementary file 1 [file ijms-24-10670-s001.zip › ijms-2443350-supplementary.pdf]

Table S1. 48 heterozygous, uncertain pathogenic character, frequent or rare variants in *C2orf71*. Silent mutations are not included.

Nucleotide change	Amino acid change	In silico prediction (P/SS / SIFT)	1000G/EVS	Ref
c.77G>A	p.Trp26*	PoD	NA	39
c.85C>T	p.Arg29Trp	PoD / D	0.046/NA	54,57
c.158 C>T	p.Ala53Val	B / T	NA	30
c.184C>G	p.Pro62Ala	NA	NA	114
c.239A>G	p.Asp80Gly	B / T	NA	54
c.400_401del	p.Ser134*	D	NA	39
c.407A>G	p.Glu136Gly	B / T	NA	54
c.530C>T	p.Pro177Leu	PoD / D	NA	55
c.679G>A	p.Glu227Lys	B / D	NA	30,44
c.773G>T	p.Arg258Ile	PoD / T	NA	44
c.935G>A	p.Ser312Asn	B / T	NA	
c.958C>T	p.Arg320Cys	B / T	NA	
c.1114G>A	p.Asp372Asn	B / PoD	NA	
c.1132G>A	p.Glu378Lys	B / T	NA	
c.1545A>G	p.Gln515Gln	NA	NA	
c.1835T>C	p.Leu612Pro	PoD / D	NA	
-	p. Arg571-Pro576del	PoD	NA	72
c.1844T>A	p.Val615Asp	PoD / T	NA	30,44
c.1882G>A	p.Ala628Thr	B / T	NA	44
c.1886dup	p.Ala631Cysfs*49	PoD	NA	39
c.1999A>T	p.Lys667*	PoD	NA	39
c.2063G>A	p.Cys688Tyr	PoD / T	NA	44
c.2485C>T	p.His829Tyr	B / T	NA	
c.2498C>T	p.Pro833Leu	PoD / D	NA	
c.2502T>C	p.Pro834Pro	NA	NA	
c.2600C>T	p.Pro867Leu	B / T	NA	44,114
c.2630G>A	p.Trp877*	PoD	NA	39
c.2672T>C	p.Leu889Pro	NA	NA	72
c.2860C>T	p.Pro954Ser	PoD / T	NA	44
c.2864G>A	p.Arg955Gln	B / T	NA	114
c.2875G>A	p.Ala959Thr	B / T	NA	44,114
c.2889C>T	p.Ser963Ser	NA	NA	54
c.2918C>G	p.Ser973*	PoD	NA	39
c.2965C>G	p.Pro989Ala	B / T	0.366/0.0083	54
c.3058_3059del CAinsAG	p.Gln1020Arg	B / T	NA	44,114
c.3277A>G	p.Met1093Val	B / T	NA/0.0077	54
c.3291G>A	p.Gln1097Gln	NA	NA	44
c.3527A>G	p.Gln1176Arg	PoD / T	0.183/NA	54
c.3530G>A	p.Arg1177Gln	B / T	NA	44
c.3447G>A	p.Pro1149Pro	NA	NA	
c.3478G>A	p.Ala1160Thr	B / T	NA	
c.3522C>T	p.Asp1174Asp	NA	NA	
c.3581C>T	p.Ala1194Val	NA	NA	115
c.3626A>G	p.Asp1209Gly	PoD / T	NA	30
c.3722C>T	p.Ser1241Phe	B / D	NA/0.0082	54
c.3672_3674dup	p.Ser1225dup	NA	NA	44
c.3739G>A	p.Gly1247Ser	B / T	NA	44,114
c.3803G>A	p.Arg1268Gln	NA	NA	116
c.3840G>A	p.Ala1280Ala	NA	NA	44

P/SS; Polyphen-2/Splice Site Prediction; PoD, possibly damaging; B, benign; D, damaging; T, tolerated; NA, not available.
